# Low-Dose Ionizing Radiation-Crosslinking Immunoprecipitation (LDIR-CLIP) Identified Irradiation-Sensitive RNAs for RNA-Binding Protein HuR-Mediated Decay

**DOI:** 10.3390/biology12121533

**Published:** 2023-12-15

**Authors:** Ji Won Lee, Hyejin Mun, Jeong-Hyun Kim, Seungbeom Ko, Young-Kook Kim, Min Ji Shim, Kyungmin Kim, Chul Woong Ho, Hyun Bong Park, Meesun Kim, Chaeyoung Lee, Si Ho Choi, Jung-Woong Kim, Ji-Hoon Jeong, Je-Hyun Yoon, Kyung-Won Min, Tae Gen Son

**Affiliations:** 1 Department of Biology, College of Natural Sciences, Gangneung-Wonju National University, Gangneung-si 25457, Republic of Korea; wldnjs1502@gmail.com (J.W.L.); slk092767@gmail.com (M.J.S.); kimkmin98@gwnu.ac.kr (K.K.); hochw1216@gmail.com (C.W.H.); hyunbong.park@gwnu.ac.kr (H.B.P.); 2 Department of Biochemistry and Molecular Biology, Medical University of South Carolina, Charleston, SC 29425, USA; hyejin-mun@ouhsc.edu (H.M.); brianko811@gmail.com (S.K.); jehyun-yoon@ouhsc.edu (J.-H.Y.); 3 Department of Oncology Science, University of Oklahoma, Oklahoma City, OK 73104, USA; jihoon-jeong@ouhsc.edu; 4 Department of Medicine, University of Ulsan College of Medicine, Seoul 05505, Republic of Korea; soykm@naver.com; 5 Biomedical Sciences Graduate Program (BMSGP), Chonnam National University, Hwasun 58128, Republic of Korea; ykk@chonnam.ac.kr; 6 Department of Biochemistry, Chonnam National University Medical School, Hwasun 58128, Republic of Korea; 7 Research Center, Dongnam Institute of Radiological and Medical Science, Busan 46033, Republic of Korea; mskim@dirams.re.kr (M.K.); get3232@dirams.re.kr (C.L.); sihochoi@dirams.re.kr (S.H.C.); 8 Department of Life Science, College of Natural Sciences, Chung-Ang University, Seoul 06974, Republic of Korea; jungkim@cau.ac.kr

**Keywords:** low-dose ionizing radiation (LDIR), RNA crosslinking, post-transcriptional gene regulation

## Abstract

**Simple Summary:**

This study addresses the limited understanding of the molecular consequences of low-dose ionizing radiation (LDIR), a relatively unexplored area compared to high-dose radiation methods. The research focuses on profiling RNA species crosslinked to the RNA-binding protein human antigen R (HuR) using LDIR and high throughput RNA sequencing. The developed method involves isolating RNA fragments crosslinked to HuR through LDIR and immunoprecipitation followed by RNA sequencing, revealing rapid degradation of target mRNAs (e.g., *PAX6*, *ZFP91*, *NR2F6*, and *CAND2*) in human cell lines. Notably, the downregulation of *PAX6* and *NR2F6* is identified as a protein mediating the beneficial effects of LDIR on cell viability. The findings underscore the significance of investigating post-transcriptional gene regulation under LDIR conditions. Overall, this research introduces a valuable approach for understanding the impact of LDIR on RNA dynamics, shedding light on the molecular consequences and potential therapeutic applications of low-dose ionizing radiation.

**Abstract:**

Although ionizing radiation (IR) is widely used for therapeutic and research purposes, studies on low-dose ionizing radiation (LDIR) are limited compared with those on other IR approaches, such as high-dose gamma irradiation and ultraviolet irradiation. High-dose IR affects DNA damage response and nucleotide–protein crosslinking, among other processes; however, the molecular consequences of LDIR have been poorly investigated. Here, we developed a method to profile RNA species crosslinked to an RNA-binding protein, namely, human antigen R (HuR), using LDIR and high-throughput RNA sequencing. The RNA fragments isolated via LDIR-crosslinking and immunoprecipitation sequencing were crosslinked to HuR and protected from RNase-mediated digestion. Upon crosslinking HuR to target mRNAs such as *PAX6*, *ZFP91*, *NR2F6*, and *CAND2*, the transcripts degraded rapidly in human cell lines. Additionally, *PAX6* and *NR2F6* downregulation mediated the beneficial effects of LDIR on cell viability. Thus, our approach provides a method for investigating post-transcriptional gene regulation using LDIR.

## 1. Introduction

Understanding the biological effects of low-dose ionizing radiation (LDIR) is important, as healthcare workers and the general public are exposed to varied levels of radiation [1]. Toward this end, in vitro studies using humans, non-human animals, and cloned cells have been conducted. However, the biological effects of LDIR remain unclear, as direct means of evaluating or understanding these effects are unavailable [2,3]. In humans, IR induces the production of reactive oxygen species (ROS), which induce genotoxic stress and alter DNA damage reactions, enabling its application in the interventional radiology of cancer [4,5]. High-dose IR causes DNA lesions, indicating that many proteins act on the DNA to alter important cellular events such as cell cycle regulation, signaling, replication, transcription, and DNA repair [6,7,8,9]. RNA-binding proteins (RBPs) have recently been proposed as new players in DNA damage response by acting on the chromatin region [10,11,12]. However, transcriptional response in conjunction with DNA repair proteins and LDIR-mediated post-transcriptional gene regulation by RBPs has not been studied.

Human antigen R (HuR)—an RBP—acts as a survival factor in IR-induced (10 Gy/various time points) DNA damage by modulating the translation of a subset of target mRNAs [13]. However, the impact of low dose rate IR (<5 Gy/h) on HuR-mediated post-transcriptional gene regulation needs to be explored using a transcriptome-wide approach. Ultraviolet (UV) crosslinking and immunoprecipitation (CLIP) with high-throughput sequencing—the most widely used transcriptomic approach for mapping RNA-protein interaction sites—has opened new avenues for understanding the complex interaction network between RBPs and RNAs within the cell [14,15,16]. During the last decade, several derivatives of CLIP-based methods have emerged. For instance, photoactivable ribonucleoside-enhanced CLIP (PAR-CLIP) has been developed, incorporating photoactivatable modified nucleoside analogues such as 4-thiouridine to enhance crosslinking precision and efficiency [17,18,19,20]. Most CLIP-derivative methods rely on irradiating cells with UV light to induce covalent bond formation between proteins and directly associated RNAs. Considering that physical inducers of crosslinking, such as UV light, can induce molecular crosslinking between RBPs and RNAs, we aimed to investigate whether IR could induce crosslinking between RNAs and proteins, revealing its contribution to post-transcriptional gene regulation [21].

Here, we used LDIR-CLIP sequencing to capture the HuR-RNA interactome to identify HuR target mRNAs under LDIR exposure in order to understand the biological effects of LDIR in pathophysiological conditions such as cancer. Our results revealed that LDIR did not affect HuR localization or phosphorylation; however, the stability of the HuR protein following 12 h irradiation at 0.05 Gy was correlated with decreased stability of the HuR target mRNAs. These findings indicate that LDIR exposure for a relatively long duration may exert biological effects.

## 2. Methods

### 2.1. Cell Culture, Plasmids, Transfection, and Irradiation

HEK293 cells were obtained from ATCC (Manassas, VA, USA) and cultured following the manufacturer’s instructions. Cas9- and sgRNA-expressing vectors pX458 (Addgene #48138) were constructed to generate HuR KO HEK293 cells. pEGFPC2-*Pax6* and pDC316-EGFP-*mNR2F6* were used to overexpress *PAX6* and *NR2F6*, respectively. pDC316-EGFP-*mNR2F6* plasmid DNA was kindly provided by Yan Lu from Fudan University. Plasmid DNAs were transfected into HEK293 and HuR KO HEK293 cell lines using Lipofectamine 2000 (Lipofectamine2000, #MAN0007824, Invitrogen, a subsidiary of Thermo Fisher Scientific, Carlsbad, CA, USA). The irradiation was conducted using a ^137^Cs irradiation source obtained from Eckert & Ziegler, Berlin, Germany. The γ-rays used for irradiation were emitted from the ^137^Cs source. Dosimetry methodology involved the regular calibration of the radiation dose using glass dosimeters. The dosimeters were calibrated on a monthly basis to ensure accuracy and reliability in measuring the radiation dose. HEK293 and HuR KO HEK293 cells were exposed to 0.05 Gy of γ-rays from a ^137^Cs irradiation source (Eckert & Ziegler, Berlin, Germany) at a dose rate of 0.22 mGys^−1^. The transfected cells were harvested 24 h after irradiation.

### 2.2. Generation of Human HuR Knockout Cell Lines

We employed the pX458 plasmid for the construction of CRISPR/Cas9 constructs transiently expressing three sgRNAs targeting HuR and wild type SpCas9. The pX458 plasmid was linearized with BbsI before insertion of the sgRNAs. Cloning was carried out using the DNA primers listed in Supplementary Table S1. Genomic DNA was extracted using the Wizard^®^ Genomic DNA Purification Kit (Promega Corporation, Madison, WI, USA) and amplified with Phusion polymerase (New England Biolabs, Ipswich, MA, USA). The corresponding primers spanned approximately 80 bp upstream to 80 bp downstream of the CRISPR-Cas9 cleavage site. The first round of PCR amplification utilized a 100 ng template per sample. Purification of PCR products was performed using a QIAquick Gel Extraction Kit (Qiagen, a subsidiary of Thermo Fisher Scientific, Waltham, MA, USA). For the second round of PCR, 20 ng of the purified PCR products from the first round were annealed with Illumina adapter and barcode sequences. The primers for this PCR step are listed in Supplementary Table S1. The resulting products underwent isolation, purification, and mixing, then were subjected to 150 paired-end sequencing cycles on the HiSeq system (Illumina, San Diego, CA, USA).

Approximately 20,000 cells were seeded in six-well plates containing 3 mL Opti-MEM without penicillin/streptomycin and incubated at 37 °C for 24 h. For HuR knockout, 1 μg HuR CRISPR/Cas9 KO plasmid was added to the transfection media to a final volume of 150 μL (solution A). Additionally, 10 μL of Lipofectamine 2000 (Lipofectamine™ 2000, #MAN0007824, Invitrogen) was added to the transfection media to a final volume of 150 μL (solution B). After 5 min, solution A was added to solution B and incubated at 22 °C for 10 min. Afterwards, the mixture was added to each well and the cells were further incubated for 24 h before sorting.

Subsequently, the transfected cells were washed, rinsed with ice-cold phosphate buffer saline, and trypsinized for 5 min in an incubator. Next, the trypsinized cells were transferred into a 15 mL tube and centrifuged at 1200 rpm for 5 min. The resulting pellets were resuspended in 1 mL culture medium, then green fluorescent protein-positive HuR knockout cell populations were sorted into 3 mL culture media using a FACS Aria cell sorter (BD, Franklin Lakes, NJ, USA). Finally, the cells were analyzed for indel frequency and western blotting was conducted.

### 2.3. MTS Assays

Irradiated cells were seeded into 96-well plates (2 × 10^3^ cells/well; in four replicates) and cell proliferation was quantified using an MTS Assay Kit (CellTiter 96^®^ AQueous One Solution Cell Proliferation Assay, #G3580; Promega Corporation) following the manufacturer’s instructions. First, approximately 40 μL MTS solution was added to 200 μL culture media with cells after days 0, 1, 3, and 6 and the mixture was incubated at 37 °C for 1 h. Next, cell viability was measured at 492 nm using a Tristar3 Multimode Reader (Berthold Technologies, Bad Wildbad, Germany) and the results were analyzed using MikroWin software 2010.

### 2.4. Western Blotting

Total proteins were lysed from cells irradiated on day 6 using ice-cold lysis buffer (PRO-PREP™ Protein Extraction Solution, #17801, iNTRON Biotechnology, Seongnam-si, Gyeonggi-do, Republic of Korea) containing a protease inhibitor (Protease Inhibitor Cocktail powder, #P2714, Sigma-Aldrich, a subsidiary of Merk KGaA, Darmstadt, Germany). Next, the lysed protein concentration was measured using BCA reagents (Pierce™ BCA Protein Assay Kit, #23227, Thermo Fisher Scientific) and proteins were resolved using 4–12% gradient SDS-PAGE (Bolt™ Bis-Tris Plus Gels, #MAN0007902, Invitrogen). Afterwards, they were transferred onto PVDF membranes (Immobilo-P, #IPVH00010, MilliporeSigma, Burlington, MA, USA) using a semi-dry electroblotting system (Power Blotter System, #PB0012 and #PB0013, Invitrogen). Next, the membranes were blocked with 5% non-fat milk or BSA in TBST and probed using primary antibodies against HuR (ms, Santa Cruz Biotechnology Inc., Santa Cruz, CA, USA, #sc-5261), phospho-HuR [22], NR2F6 (ms, Proteintech, Deerfield, IL, USA, #60117-2-lg), PAX6 (rb, Invitrogen, #42-6600), Beta-actin (ms, Santa Cruz Biotechnology Inc., #sc-47778), and GAPDH (ms, Santa Cruz Biotechnology Inc., #sc-47724). The unbound primary antibodies were washed with TBST and the membranes were incubated with secondary antibodies at room temperature (22 °C) for 1 h. Lastly, the protein bands were analyzed using an ECL reagent (ECL Select western blotting Detection Reagent, #RPN2235, Amersham, a subsidiary of GE Healthcare, Waukesha, IL, USA) and a gel documentation system (ImageQuant800, Amersham, UK).

### 2.5. Phos-Tag SDS-PAGE Electrophoresis

HuR phosphorylation was measured by supplementing SDS-polyacrylamide gels (10%) with Phos-tag AAL solution (Wako, Tokyo, Japan, #304-93525) following the manufacturer’s recommendations. Next, the gels were transferred to a nitrocellulose membrane using a transfer buffer containing 0.1% SDS, followed by a standard western blotting procedure.

### 2.6. LDIR-CLIP-Sequencing Analysis

For HuR LDIR-CLIP, we irradiated HEK293 cells with IR (0.02–5 Gy) to induce RNA and protein cross-linking. Next, the cells were lysed with the lysis buffer (20 mM Tris-HCl pH 7.5, 100 mM KCl, 5 mM MgCl_2_, 0.5% NP-40). The cell lysates were treated with 1 U/mL RNase T1 (Fermentas, a subsidiary of Thermo Fisher Scientific) and immunoprecipitated with HuR antibody (#sc-5261). Afterwards, the RNA fragments bound to Protein G Sepharose beads were trimmed using 100 U/mL RNase T1. The beads were then washed with the lysis buffer and resuspended in dephosphorylation buffer. The protein-RNA complexes were purified from polyacrylamide gel. Subsequently, the proteins were digested with 0.2 mg/mL proteinase K in the corresponding buffer (Roche, Basel, Switzerland). Lastly, RNA was precipitated using acidic phenol/chloroform and ethanol, then converted into the cDNA library for sequencing on an Illumina platform. The processed reads were aligned to the reference genome (GRCh37/hg19) using the Bowtie algorithm (0.12.7).

### 2.7. Ribonucleoprotein Immunoprecipitation

Total cells were lysed in lysis buffer (20 mM Tris-HCl pH 7.5, 100 mM KCl, 5 mM MgCl_2_, 0.5% NP-40) on ice for 10 min and centrifuged at 13,000 rpm at 4 °C for 15 min. Next, the lysates (1–2 mg) were incubated with 1 μg antibody against HuR (#sc-5261) or control IgG (#sc-2025) at 4 °C overnight and incubated with protein A/G Sepharose beads (Santa Cruz, #sc-2003) at 4 °C with gentle rotation for 2 h. Finally, the beads were washed five times with NT2 buffer (50 mM Tris-HCl, pH 7.5, 150 mM NaCl, 1 mM MgCl_2_, and 0.05% NP-40) and RNA isolated from the immunopellets via acidic phenol extraction was subjected to RT-qPCR.

### 2.8. RNA-Seq

HEK293 cells were exposed to LDIR at 0.05 Gy for specified durations, and total RNA was extracted from these cells for subsequent RNA sequencing. We followed the standard protocol of Illumina sequencing using the TruSeq Stranded Total RNA kit with Ribo-Zero (Part#15031048 Rev. E) and NovaSeq6000 S4 (San Diego, CA, USA, 150 bp Paired-End, PE). Analyses were performed on two paired-end samples. Trimmed reads were mapped to the reference genome (Homo sapiens (Human) Genome) with hierarchical indexing for spliced alignment of transcripts. After the read mapping, StringTie was used for transcript assembly. The expression profile was calculated for each sample and transcript/gene as the read count and fragment per kilobase of transcript per million mapped reads.

### 2.9. mRNA Stability Assay

Half-lives of the HuR target mRNAs were measured using RT-qPCR. After 6 or 12 h incubation following 0.05 Gy irradiation, the cells were treated with actinomycin D (2.5 μg/mL) to inhibit transcription. Finally, RNA was extracted at various time points to measure the levels of the HuR target mRNAs and *GAPDH* mRNA and calculate their half-lives.

### 2.10. Quantitative RT-PCR Analysis

Total RNA was extracted from cells using TRIzol (Thermo Fisher Scientific, #15596026) following the manufacturer’s protocol. Briefly, 1 μg total RNA was reverse-transcribed using random hexamer oligonucleotides and reverse transcriptase (Thermo Fisher Scientific (#EP0753). Real-time PCR was performed using SYBR Green (Thermo Fisher, #A25918) on a QuantStudio3 real-time PCR detection system. The primers used in this study are listed in Supplementary Table S1.

### 2.11. Statistical and Reproducibility Analysis

Statistical analyses were performed using GraphPad Prism software 9. Comparisons between the two groups were performed using Student’s *t*-test. All experiments were repeated at least twice. All error bars represent the means ± standard deviation (SD).

## 3. Results

### 3.1. LDIR Promotes Crosslinking of RNA-Binding Protein HuR with Its Target RNAs

**Figure 1:**
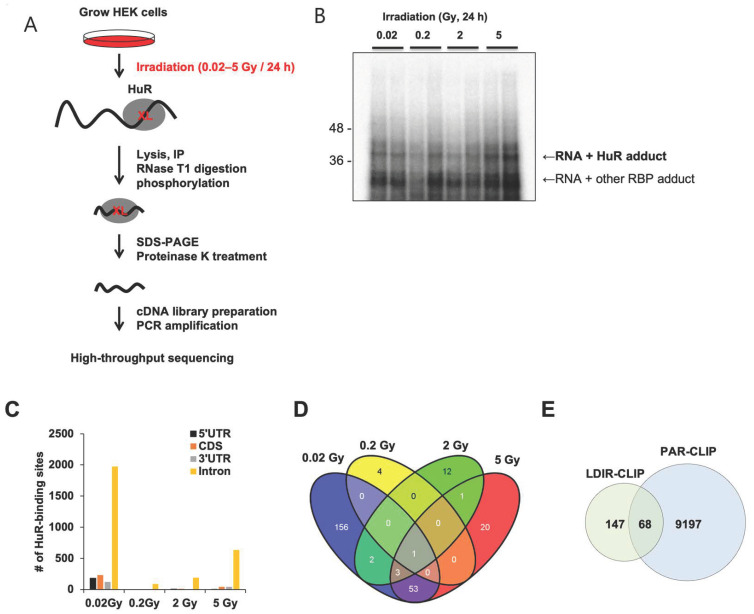
A subset of RNAs targeted by the RNA-binding protein HuR was identified using the LDIR-CLIP method. (**A**) Schematic diagram of LDIR-CLIP-sequencing analysis. (**B**) Autoradiograph of RNA fragments crosslinked with HuR upon irradiation at doses of 0.02–5 Gy for 24 h. Two loadings at each dose are biological replicates, and then the samples were pooled together for a single library preparation and high-throughput sequencing (*n* = 1). (**C**) Number of HuR-binding sites on CDS, UTR, and introns following irradiation at 0.02–5 Gy for 24 h. (**D**) Venn diagrams summarizing the transcripts associated with HuR following irradiation at 0.02–5 Gy for 24 h. (**E**) Number of transcripts commonly identified using LDIR-CLIP and PAR-CLIP. The original blot image is presented in Supplementary Figure S4.

To profile transcripts crosslinked with RBP HuR (Figure 1A), we irradiated human embryonic kidney (HEK) 293 cells at doses of 0.02–5 Gy. After 24 h irradiation, the cells were lysed and centrifuged and the supernatant was collected. After HuR immunoprecipitation and RNase T1 digestion, the protected RNA fragments were phosphorylated and subjected to SDS-PAGE. Autoradiography of RNA fragments crosslinked with HuR was performed to determine the complex size using SDS-PAGE (Figure 1B). Next, the complex was isolated and treated with proteinase K to purify RNAs that were then used for cDNA library preparation, PCR amplification, and high-throughput RNA sequencing.

HuR LDIR-CLIP sequencing identified RNA fragments crosslinked to HuR and protected from RNase T1-mediated digestion. The largest number of independent RNA fragments was profiled after 24 h irradiation at doses of 0.02–5 Gy. In the 0.02 Gy-irradiated samples, 179 instances of crosslinking occurred in the 5′ untranslated region (UTR) across 21 transcripts, 231 in the codon-determining sequences (CDS) across 29 transcripts, 120 in the 3′ UTR across 11 transcripts, and 1986 in the intronic sequences across 191 transcripts. (Figure 1C, Supplementary Table S2). Moreover, 53 common transcripts were isolated after 0.02 and 5 Gy irradiation (Figure 1D) and 68 transcripts overlapped with HuR PAR-CLIP, which was 0.9% of the total reads from the previous PAR-CLIP analysis [15] (Figure 1E, Supplementary Table S3). These results demonstrate that LDIR promotes HuR crosslinking with RNAs in a dose-dependent manner.

**Figure 2:**
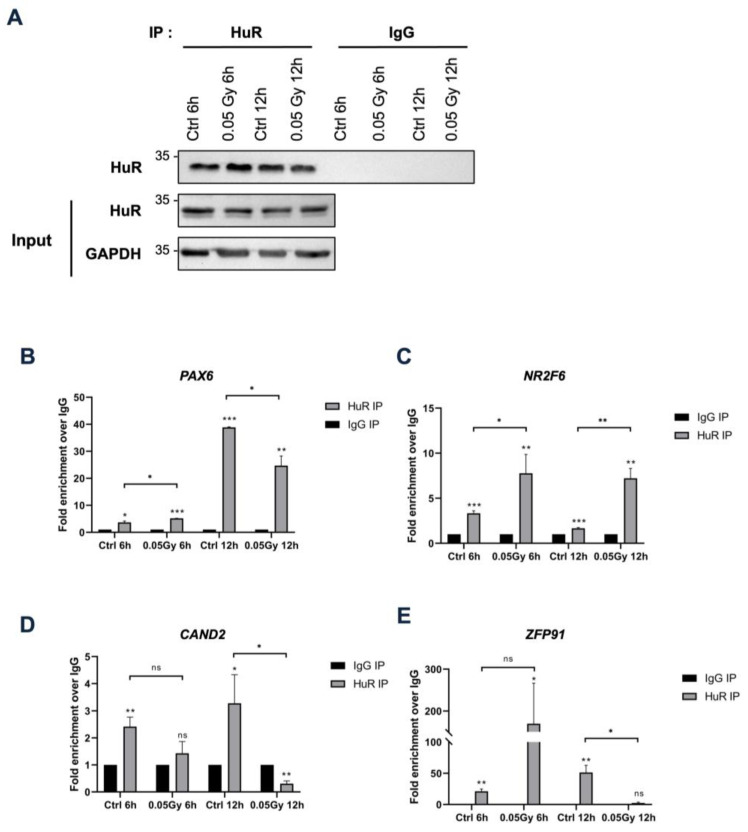
LDIR affects the binding of HuR with target mRNAs. (**A**) Immunoprecipitation efficiencies were analyzed using HuR antibody after immunoprecipitation. (**B**–**E**) HuR RIP RT-qPCR of RNAs profiled in LDIR-CLIP-seq. Data represent the mean ± SD from *n* = 2 independent experiments. *: *p* < 0.05; **: *p* < 0.01, ***: *p* < 0.001, ns: *p* > 0.05 from Student’s *t*-test. Original blot images are presented in Supplementary Figure S4.

To verify crosslinking of HuR and bound transcripts, we selected transcripts containing HuR-bound sequences in their CDS and UTRs, owing to the roles of HuR in target mRNA stability, localization, and translation. We focused on transcriptional regulators that broadly affect gene expression in response to LDIR. *PAX6* (5′ UTR), *NR2F6* (5′ UTR), *CAND2* (5′ UTR), and *ZFP91* (5′ UTR and CDS) were exclusively detected in the 0.02 Gy group following LDIR-CLIP sequencing and HuR PAR-CLIP. We performed experiments with various doses for LDIR-CLIP, however, we settled on 0.05 Gy, as we observed a decrease in the overall number of HuR-binding sites on target mRNAs after irradiation at doses greater than 0.2 Gy, in contrast to the observed effects at 0.02 Gy for 24 h in HEK293 cells (Figure 1D, Supplementary Table S2). We reasoned that 0.02 Gy might represent an extremely low level of irradiation, and that a 24 h duration might impose extreme conditions. Therefore, we opted for 0.05 Gy for 6 h as short-term exposure and for 12 h as long-term exposure for the subsequent experiments. Enrichment of these transcripts in HuR immunopellets irradiated at 0.05 Gy was analyzed by HuR RIP-qPCR analysis, although the relative enrichment of the transcripts varied irrespective of 0.05 Gy irradiation in HEK293 cells (Figure 2B–D). Additionally, we confirmed the successful immunoprecipitation of HuR from each sample (Figure 2A). Therefore, LDIR-CLIP sequencing is a tool for investigating the biochemical interactions between HuR and target mRNAs in human cell lines.

### 3.2. LDIR Modulates the Stability of HuR-Crosslinked mRNAs

**Figure 3:**
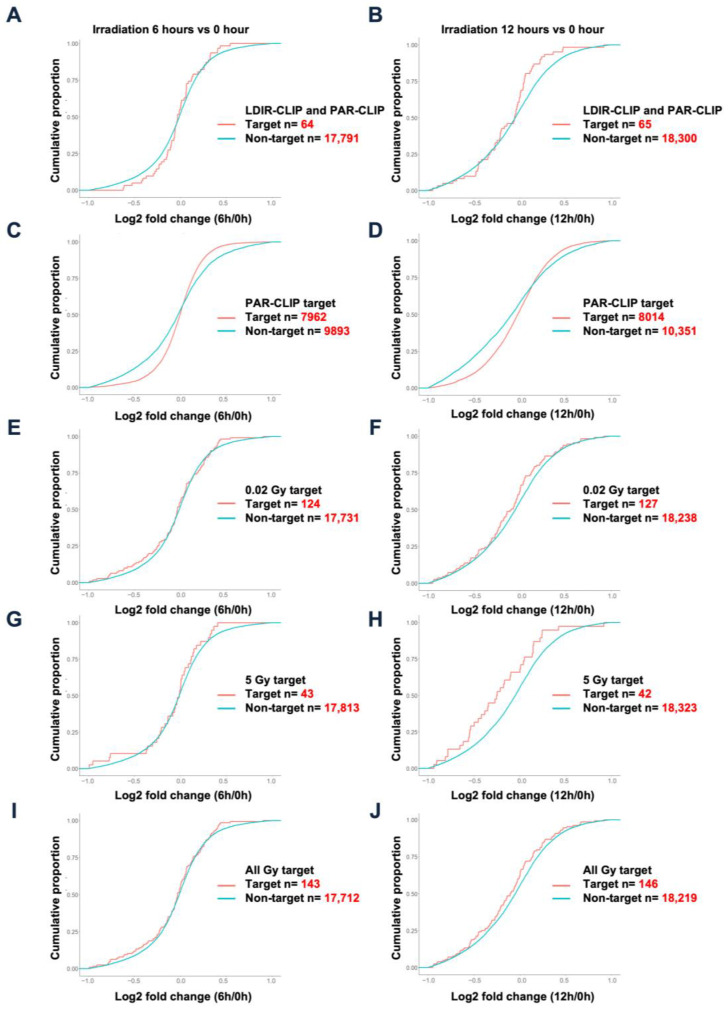
Comparison of LDIR-CLIP and PAR-CLIP regarding altered gene expression upon LDIR: (**A**,**C**,**E**,**G**,**I**) cumulative distribution function (CDF) plots of transcripts targeted by HuR after 6 h irradiation and (**B**,**D**,**F**,**H**,**J**) CDF plots of transcripts targeted by HuR after 12 h irradiation.

**Figure 4:**
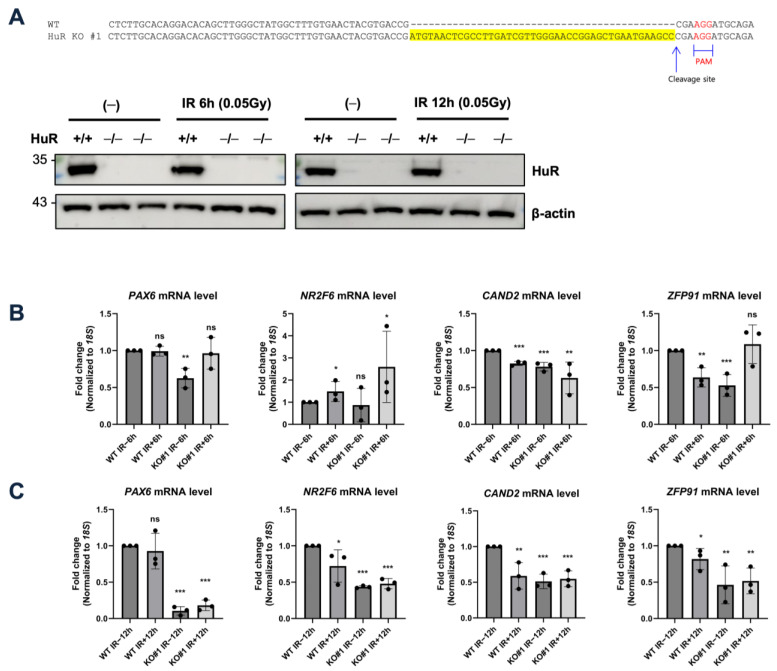
Changes in levels of HuR-targeted RNA following LDIR. (**A**) (**Top**) Schematic diagram of deep-sequencing data showing the genomic locus with CRISPR guide sequence (yellow), PAM motif (red), and predicted cleavage site (arrow). (**Bottom**) Western blot analysis of HEK293 cells expressing the sgRNA targeting the control region or *HuR* gene. (**B**,**C**) RT-qPCR to detect *PAX6*, *NR2F6*, *CAND2*, and *ZFP91* expression in HuR^+/+^ and ^−/−^ cells upon 0.05 Gy irradiation for 6 h (**B**) or 12 h (**C**). Data represent the mean ± SD from *n* = 3 independent experiments. *: *p* < 0.05; **: *p* < 0.01, ***: *p* < 0.001, ns: *p* > 0.05 from Student’s *t*-test. Original blot images are presented in Supplementary Figure S4.

To evaluate the consequences of HuR and its target mRNA interaction upon LDIR exposure, we generated high-throughput RNA sequencing datasets using the total RNA isolated from HEK293 cells with or without exposure to 0.05 Gy irradiation after 6 or 12 h (Supplementary Table S4). Cumulative distribution function (CDF) plots were generated by dividing a group of transcripts profiled using PAR-CLIP analysis [15] and/or LDIR-CLIP sequencing to determine changes in HuR target RNA expression (Figure 3). We observed similar patterns of HuR target RNAs using LDIR-CLIP and PAR-CLIP (Figure 3A,B). Moreover, 0.05 Gy irradiation stabilized the HuR target RNAs detected by PAR-CLIP, shifting the cumulative distribution function slightly to the right after 12 h compared with that after 6 h (Figure 3C,D). We selected HuR target RNAs from LDIR-CLIP following 0.02 Gy irradiation (Figure 1E) from the total RNA-seq. Our results revealed that 0.02 Gy irradiation slightly reduced the expression of a subset of HuR target RNAs detected using LDIR-CLIP (Figure 3E,F). Similarly, 5 Gy irradiation reduced the expression of a subset of HuR target RNAs detected using LDIR-CLIP (Figure 3G,H). Comparison of the target RNAs obtained after treatment with all the tested doses of irradiation to the non-target RNAs showed that LDIR decreased the expression of the HuR target RNAs at 6 and 12 h exposure to LDIR (Figure 3I,J), indicating that LDIR reduced the expression of a subset of HuR target RNAs detected using LDIR-CLIP in HEK293 cells. This prompted us to hypothesize that LDIR negatively impacts the expression of HuR target genes (Figure 4).

**Figure 5:**
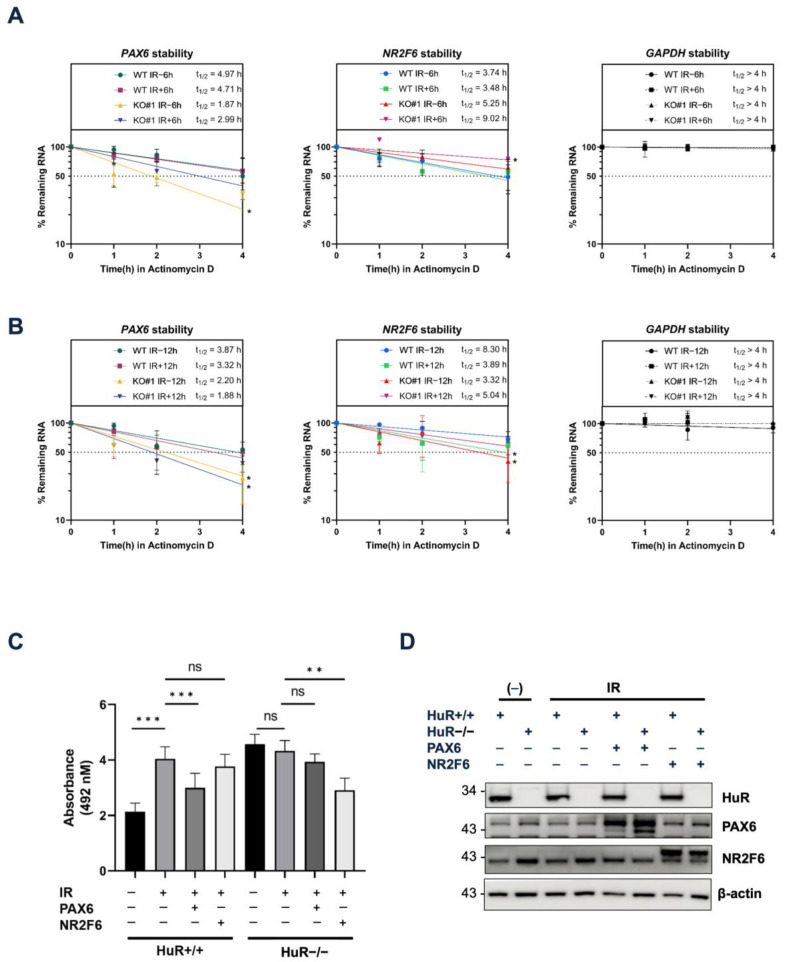
LDIR destabilizes HuR-bound mRNAs to enhance cell viability. (**A**,**B**) mRNA stability of *PAX6*, *NR2F6*, and *GAPDH* mRNAs following 0.05 Gy irradiation for 6 h (**A**) or 12 h (**B**) in HuR^+/+^ and ^−/−^ cells. (**C**) Viability of HuR^+/+^ and ^−/−^ cells upon 0.05 Gy irradiation at 6 days when *PAX6* or *NR2F6* was re-introduced. (**D**) HuR KO, *PAX6*, or *NR2F6* overexpression was confirmed using western blot analysis. Beta-actin was used as an endogenous control. Data represent the mean ± SD from *n* = 4 independent experiments. *: *p* < 0.05, **: *p* < 0.01, ***: *p* < 0.001, ns: *p* > 0.05 from Student’s *t*-test. Original blot images are presented in Supplementary Figure S4.

Additionally, we conducted differential expression gene analysis using total RNA seq after 0.05 Gy irradiation for 6 or 12 h to evaluate the characteristics of the altered genes (Supplementary Figure S1A,B). The results revealed that longer exposure (12 h) to 0.05 Gy reduced the levels of many mRNA compared to a short exposure (6 h) (Supplementary Figure S1A,B). This observation is consistent with the findings in Figure 3, Figure 4 and Figure 5, suggesting that prolonged low-dose exposure may exert biological effects. As suggested, molecular functions were analyzed using Gene Ontology (GO) analysis on differentially expressed genes. The affected molecular functions by LDIR were primarily related to DNA-binding after 6 h exposure and growth factor binding and activity after 12 h irradiation. These results suggest that LDIR potentially contributes to diverse biological processes and gene expression regulation (Supplementary Figure S1C,D).

Next, we generated a HuR KO cell line using the CRISPR/Cas9 system to investigate whether LDIR-mediated repression of gene expression was HuR-dependent (Figure 4A and Supplementary Figure S2A). Although we did not observe significant changes following 6 h irradiation at 0.05 Gy (Figure 4B), 12 h exposure to 0.05 Gy irradiation reduced the steady-state expression levels of HuR target genes, consistent with the observation that HuR KO considerably decreased the levels of HuR target mRNAs (Figure 4C). This implies that LDIR can suppress HuR activity, leading to decreased expression of HuR target genes, consistent with previous observations that HuR stabilizes its target mRNAs [23,24,25].

We next confirmed whether HuR KO and LDIR modulate the stability of HuR target mRNAs by measuring their stability. We treated HEK293 cells with actinomycin D 30 min before 0.05 Gy irradiation to shut off mRNA transcription. We harvested and lysed the cells at 0, 1, 2, and 4 h post-irradiation and performed RT-qPCR to measure the stability of these mRNAs. The stability of *GAPDH*—a HuR non-target mRNA—in HuR KO did not change significantly after 0.05 Gy irradiation. However, we observed the following: (1) 0.05 Gy irradiation in wild-type (WT) decreased the stability of HuR target mRNAs, with prolonged exposure (0.05 Gy/12 h) showing a more profound impact than 0.05 Gy/6 h, consistent with the observation that 12 h exposure decreased the steady-state levels of target mRNAs more significantly than 6 h exposure; (2) HuR KO without 0.05 Gy irradiation significantly decreased the half-life of HuR target mRNAs; and (3) HuR KO with 0.05 Gy/6 or 12 h irradiation fluctuated the stability of HuR target mRNAs depending on the incubation time (Figure 5A,B and Supplementary Figure S2B,C).

Furthermore, we observed no changes in the protein levels of HuR target mRNAs. This is because the incubation time (6 and 12 h) was insufficient to alter their translational levels. Little or no HuR was crosslinked upon LDIR, barely influencing the expression of target genes (Figure 5D), indicating that HuR increased and decreased the stability of HuR target mRNAs under normal conditions and upon 0.05 Gy irradiation, respectively.

To examine the molecular mechanisms by which HuR affects the decay of target mRNAs upon LDIR exposure, we hypothesized that LDIR influences HuR function by altering the stability of HuR target mRNAs. Cellular stress can influence the nuclear–cytoplasmic shuttling of HuR, leading to changes in the landscape of HuR target RNAs [26]. We observed no influence on the HuR subcellular location after 0.05 Gy irradiation (Supplementary Figure S3A). Notably, HuR has numerous phosphorylation residues, and post-translational modification can influence both the subcellular localization of HuR and its binding to target mRNAs [27,28,29]. We observed that the phosphorylation status of HuR was not influenced by 0.05 Gy irradiation regardless of the exposure time compared with that in the control group (Supplementary Figure S3B,C). HuR protein stability can be modulated to maintain cellular homeostasis in response to stresses such as heat shock [30]. Interestingly, HuR protein stability slightly increased with the irradiation dose compared with that in the control (Supplementary Figure S3D), suggesting that HuR levels might increase when the irradiant energy crosses a certain threshold. These results indicate that LDIR did not influence the phosphorylation and cellular distribution of HuR but could increase the stability of HuR protein.

**Figure 6:**
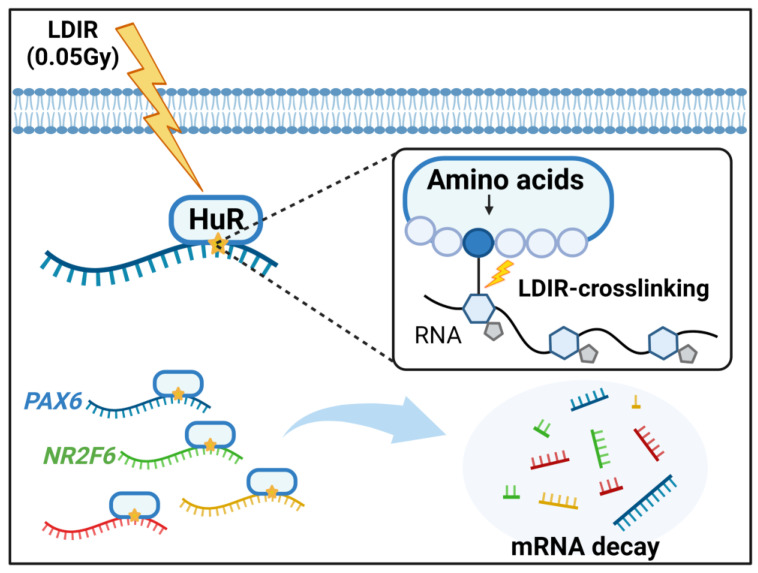
Schematic diagram of post-transcriptional gene regulation mediated by low-dose ionizing radiation (LDIR). LDIR-mediated crosslinking of *PAX6* and *NR2F6* mRNAs with HuR likely decreases their stability.

Finally, we investigated whether LDIR-influenced cell viability depended on HuR target genes. We overexpressed *PAX6* and *NR2F6*, confirmed our results using western blot analysis (Figure 5D), and measured the cell viability after 6 days of 0.05 Gy irradiation (Figure 5C). We found that 0.05 Gy irradiation increased the cell viability of WT compared with WT without irradiation. However, the cell viability of HuR KO cells subjected to 0.05 Gy irradiation did not change significantly, indicating that HuR was required to increase cell viability. Furthermore, *PAX6* overexpression in WT subjected to 0.05 Gy irradiation decreased the cell viability compared with WT (without *PAX6* overexpression) subjected to 0.05 Gy irradiation. *PAX6* overexpression in HuR KO cells subjected to 0.05 Gy irradiation did not significantly change the cell viability. This indicates that PAX6 depletion might be required for HuR-mediated cell viability after 0.05 Gy irradiation. Moreover, we observed no change in cell viability after 0.05 Gy irradiation, and *NR2F6* overexpression reduced cell viability with no change in *PAX6* overexpression in HuR KO subjected to irradiation. This confirms that *PAX6* depletion might be required for HuR-mediated cell viability after 0.05 Gy irradiation. Collectively, our results suggest that LDIR could induce covalent crosslinking between HuR and its target mRNAs, leading to destabilization of the target mRNAs (Figure 6).

## 4. Discussion

IR modulates several biological processes that in turn affect post-transcriptional gene regulation. To gain insights into the effect of LDIR on gene expression at the RNA level, we investigated changes in the HuR-bound RNA transcriptome following LDIR exposure in HEK293 cells. LDIR-CLIP sequencing showed that IR rather than UV light regulated the post-transcriptome. Moreover, the number of HuR target RNAs identified from LDIR-CLIP sequencing was much lower than the number identified from HuR PAR-CLIP.

It is plausible that LDIR produces reactive oxygen species (ROS) such as H_2_O_2_, which could contribute to HuR oxidation and RNA crosslinking stochastically with much less efficiency than UV light [31]. LDIR-induced RNA modification could alter the RNA base sequence and thereby decrease the sensitivity of RBP-RNA interactions [32,33,34]. A relatively high dose of IR (10 Gy) reportedly promotes global dissociation of the HuR-mRNA complex [13]. Consistent with this observation, we found that the overall number of HuR-binding sites on the target mRNAs was decreased after irradiation at >0.2 Gy compared to those observed at 0.02 Gy irradiation in HEK293 cells. The assembly and remodeling of RBP-RNA complexes are dynamic and influenced by time- and space-dependent stochastic fluctuations in the expression and localization of RBP. In our study, LDIR affected neither HuR localization nor phosphorylation; however, the stability of the HuR protein following 12 h irradiation at 0.05 Gy was correlated with the decreased stability of HuR target mRNAs. These findings indicate that LDIR exposure for a relatively long duration may exert biological effects.

Here, we report the role of HuR in mediating the biological effects of LDIR. In a previous study, it was found that the 3′ UTR of the p53 mRNA was associated with HuR in a UVC-dependent manner both in vitro and in vivo. RKO cells overexpressing HuR exhibited increased levels of p53. Conversely, cells with decreased HuR expression demonstrated significantly reduced p53 translation [35]. Cold-inducible RNA-binding protein (CIRP), also known as A18 hnRNP or CIRBP, is an RNA-binding protein responding to different cellular stresses and is induced specifically by cold shock. Sun et al. [36] reported decreased expression of CIRP-induced colony formation and reduced cell viability after irradiation. Additionally, cells with CIRP knockdown exhibited an increased rate of DNA damage and reduced ability to arrest the cell cycle after irradiation. Hence, in CIRP knockdown cells experiencing DNA damage induced by irradiation, the reduced DNA repair processes led to an increased rate of apoptosis.

In this study, we discovered new targets, such as *PAX6*, *ZFP91*, *NR2F6*, and *CAND2*, whose expression was controlled by HuR in response to LDIR. Studies have shown that suppressing *PAX6* expression increases cell proliferation, decreases apoptosis, and regulates cell cycle arrest in human retinoblastoma [37] and corneal epithelial cells [38]. In our study, increased cell viability in response to LDIR decreased in HuR WT cells overexpressing *PAX6*, though not in HuR KO cells. However, *NR2F6* overexpression did not consistently regulate cell viability in LDIR-exposed HuR WT cells. *NR2F6* expression reportedly regulates proliferation; in particular, *NR2F6* knockdown significantly inhibits lung cancer cells via miR-142-3p [39,40]. Therefore, we presumed that *NR2F6* RNA expression did not decrease, while *PAX6* expression increased in response to LDIR due to a difference in the regulation mechanism of these genes.

Our study has a number of limitations. First, the exact sites of LDIR-mediated HuR-RNA crosslinking were unclear. Further exploration of molecular events and factors that influence LDIR-mediated crosslinking efficiencies can help to elucidate the influence of LDIR on gene regulation. Second, the LDIR effect was similar to that observed when HuR was knocked out in terms of the decreased steady-state levels and stability of the target mRNAs, suggesting that LDIR effects could be HuR-dependent. Therefore, the underlying mechanism(s) by which mRNAs crosslink with HuR upon LDIR exposure need to be explored.

In conclusion, our findings suggest that HuR can be crosslinked with a set of target RNAs upon LDIR exposure. Furthermore, HuR target mRNAs may be degraded due to aberrant HuR-mRNA complex formation. Our approach provides a tool for studying post-transcriptional gene regulation in response to LDIR exposure.

## 5. Conclusions

This research provides comprehensive insights into the dose-dependent effects of LDIR on the interaction between HuR and its target RNAs, shedding light on the molecular mechanisms underlying HuR-mediated responses to low-dose ionizing radiation in human cells.

## Data Availability

LDIR and RNA sequencing data and the other resources generated in this study are available from the corresponding author upon request. The data are not publicly available due to ongoing projects utilizing the same data sets.

## Supplementary Materials

The following supporting information can be downloaded at https://www.mdpi.com/article/10.3390/biology12121533/s1, Figure S1: LDIR potentially contributes to changes in molecular functions and gene regulation in human cells; Figure S2: Low-dose ionizing radiation (LDIR) affects the stability of mRNAs targeted by human antigen R (HuR); Figure S3: Molecular mechanisms of how low dose ionizing radiation (LDIR) alters the regulation of human antigen R (HuR)-mediated gene expression regulation; Figure S4: Original western blot images; Table S1: Primer sequences used in this study; Table S2: HuR-LDIR-CLIP sequencing data exposed to 0.02–5 Gy irradiation for 24 h; Table S3: Comparison of HuR binding transcripts of LDIR-CLIP irradiated at 0.02 Gy with HuR target RNAs of PAR-CLIP; Table S4: Total RNA-Seq datasets irradiated at 0.05 Gy for 6 h and 12 h post-exposure.
