# Supplementary material for: Low-Dose Ionizing Radiation-Crosslinking Immunoprecipitation (LDIR-CLIP) Identified Irradiation-Sensitive RNAs for RNA-Binding Protein HuR-Mediated Decay
Source: Biology (Basel). 2023 Dec 15;12(12):1533. doi: 10.3390/biology12121533 (PMC10740889; doi:10.3390/biology12121533)
Supplement: Supplementary file 1 [file biology-12-01533-s001.zip › Supplementary_Figures_Final_121523.pdf]

Supplementary Data

Supplementary Figures

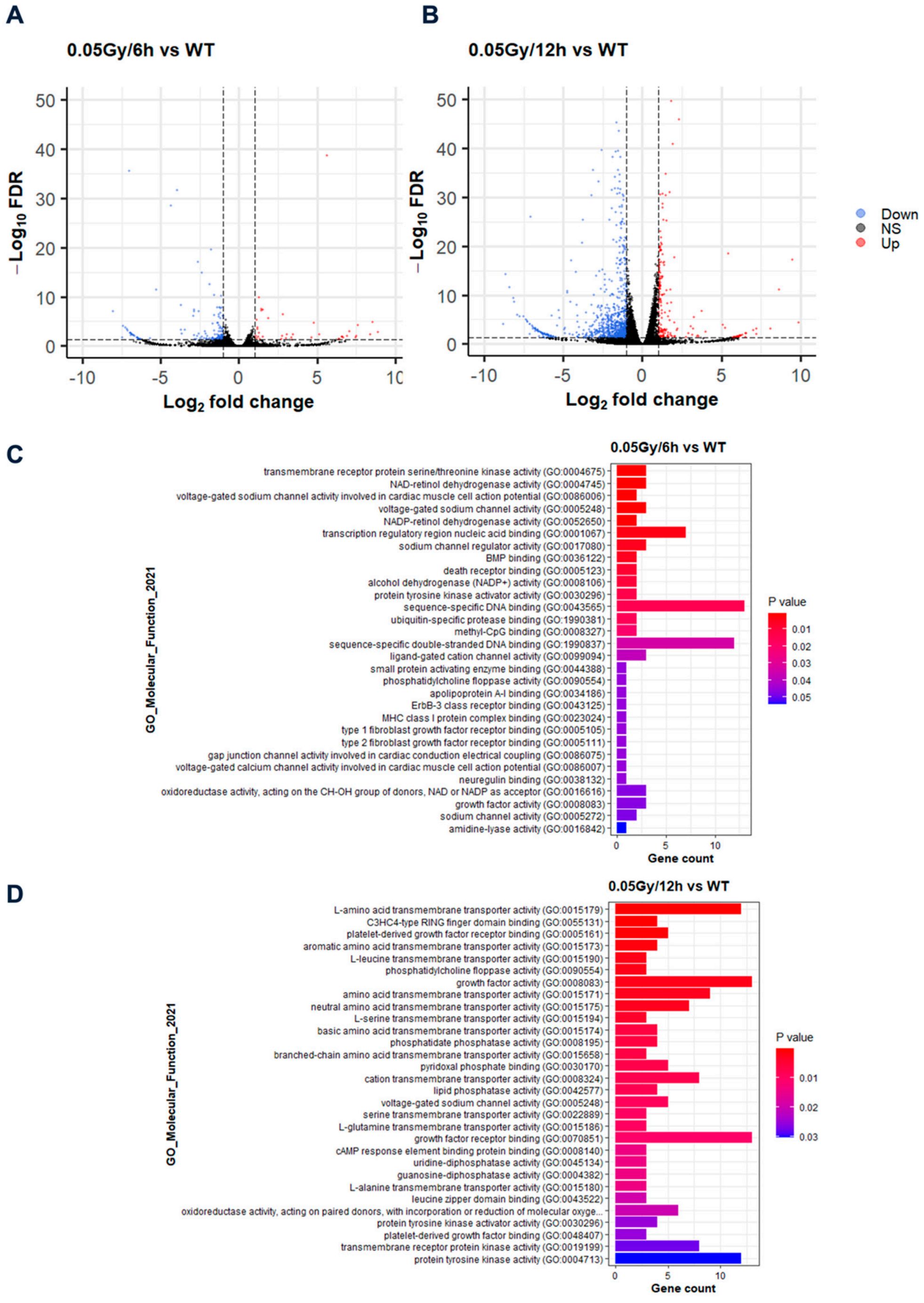

Figure S1. LDIR potentially contributes to changes in molecular functions and gene regulation in human cells. **(A-B)** Volcano plot representation of differential expression analysis of genes in 0.05 Gy irradiation for 6 h **(A)** and 12 h **(B)** versus wild type (WT). The

black dots denote non-significant, the blue dots denote downregulated genes (FDR<0.05, log2 FC<-1), and the red dots denote upregulated genes (FDR<0.05, log2 FC>1). **(C-D)** Molecular function of Gene Ontology (GO) enrichment analysis with differentially expressed genes (DEGs) in 0.05 Gy irradiation for 6 h **(C)** and 12 h **(D)**.

A

Cell lines	In-del frequency	In-del read counts	Total read counts
HuR KO #1 1	99.97%	629688	619862
HuR KO #1 2	99.89%	608450	609073

B

C

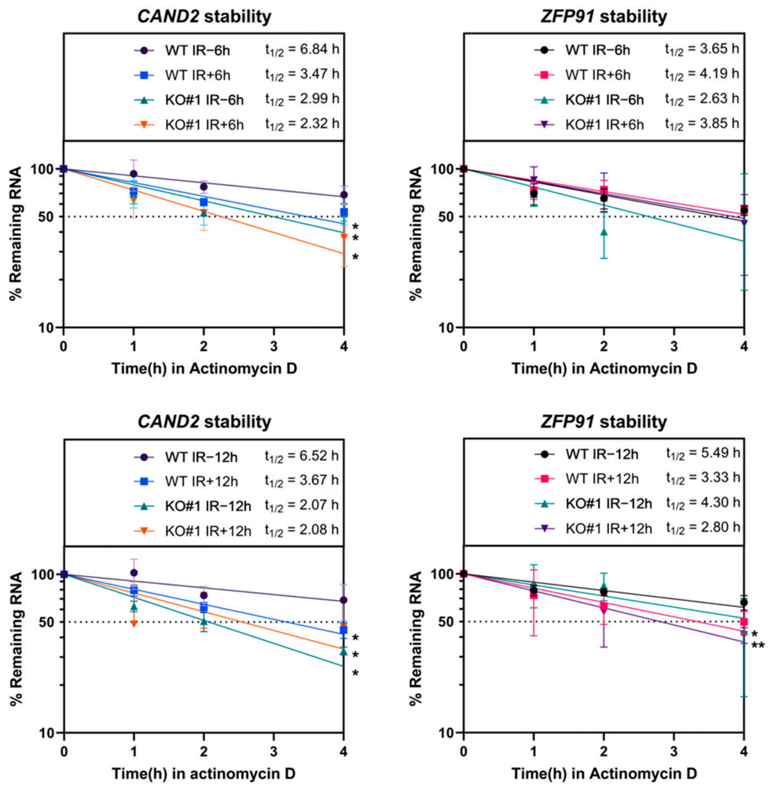

Figure S2. Low-dose ionizing radiation (LDIR) affects the stability of mRNAs targeted by human antigen R (HuR). (**A)** Indel frequencies in HuR were determined by deep sequencing using the HEK293 CRISPR/Cas9 system**. (B, C)** Stability of *CAND2* and *ZFP91* mRNAs after 0.05 Gy irradiation for 6 h **(B)** and 12 h **(C)** in HuR +/+ and -/- cells. Data represent three independent experiments. *: *p <0.05*; **: *p<0.01* from Student's *t*-test.

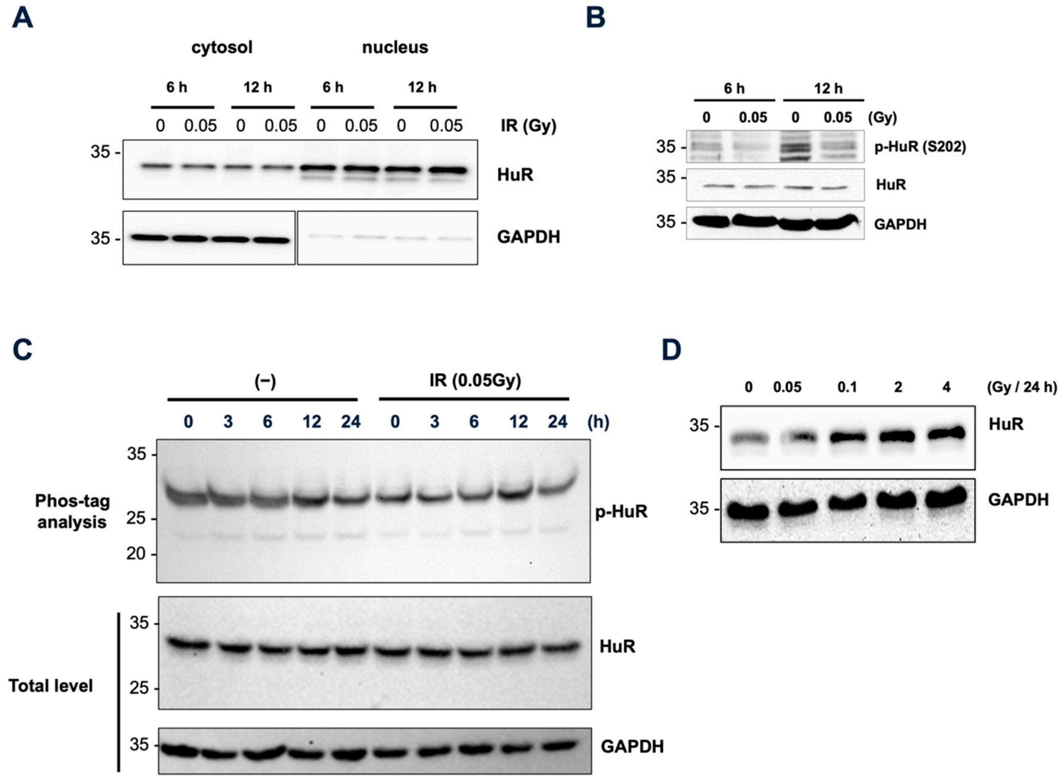

Figure S3. Molecular mechanisms of how low dose ionizing radiation (LDIR) alters the regulation of human antigen R (HuR)-mediated gene expression regulation. **(A)** Western blot analysis of the localization of HuR in cytosol and nucleus upon 0.05 Gy irradiation for 6 or 12 h. **(B)** Western blot analysis of the level of phospho HuR-S202 upon 0.05 Gy irradiation for 6 or 12 h. **(C)** Phos-tag electrophoresis demonstrating HuR phosphorylation in HEK293 cells upon 0.05 Gy irradiation for the indicated time. **(D)** Stability of HuR in HEK293 cells was detected by using western blotting after exposure to radiation for 24 h in HEK293 cells. Original blot images are presented in supplementary figure S4.
